# Micro-scale Spatial Clustering of Cholera Risk Factors in Urban Bangladesh

**DOI:** 10.1371/journal.pntd.0004400

**Published:** 2016-02-11

**Authors:** Qifang Bi, Andrew S. Azman, Syed Moinuddin Satter, Azharul Islam Khan, Dilruba Ahmed, Altaf Ahmed Riaj, Emily S. Gurley, Justin Lessler

**Affiliations:** 1 Department of Epidemiology, Bloomberg School of Public Health, Johns Hopkins University, Baltimore, Maryland, United States of America; 2 International Centre for Diarrhoeal Disease Research, Bangladesh (icddr,b), Dhaka, Bangladesh; Massachusetts General Hospital, UNITED STATES

## Abstract

Close interpersonal contact likely drives spatial clustering of cases of cholera and diarrhea, but spatial clustering of risk factors may also drive this pattern. Few studies have focused specifically on how exposures for disease cluster at small spatial scales. Improving our understanding of the micro-scale clustering of risk factors for cholera may help to target interventions and power studies with cluster designs. We selected sets of spatially matched households (matched-sets) near cholera case households between April and October 2013 in a cholera endemic urban neighborhood of Tongi Township in Bangladesh. We collected data on exposures to suspected cholera risk factors at the household and individual level. We used intra-class correlation coefficients (ICCs) to characterize clustering of exposures within matched-sets and households, and assessed if clustering depended on the geographical extent of the matched-sets. Clustering over larger spatial scales was explored by assessing the relationship between matched-sets. We also explored whether different exposures tended to appear together in individuals, households, and matched-sets. Household level exposures, including: drinking municipal supplied water (ICC = 0.97, 95%CI = 0.96, 0.98), type of latrine (ICC = 0.88, 95%CI = 0.71, 1.00), and intermittent access to drinking water (ICC = 0.96, 95%CI = 0.87, 1.00) exhibited strong clustering within matched-sets. As the geographic extent of matched-sets increased, the concordance of exposures within matched-sets decreased. Concordance between matched-sets of exposures related to water supply was elevated at distances of up to approximately 400 meters. Household level hygiene practices were correlated with infrastructure shown to increase cholera risk. Co-occurrence of different individual level exposures appeared to mostly reflect the differing domestic roles of study participants. Strong spatial clustering of exposures at a small spatial scale in a cholera endemic population suggests a possible role for highly targeted interventions. Studies with cluster designs in areas with strong spatial clustering of exposures should increase sample size to account for the correlation of these exposures.

## Author Summary

While clustering of cholera incidence had been previously described, the relative role of similar risk behaviors versus transmission dynamics is not well understood. We explored how risk factors for cholera clustered at the sub-community scale, and found significant more correlation in risk behaviors among spatially matched households than the community as a whole. We found clustering of single risk factors, and co-occurrence of different risk factors. Our results indicated that the distribution of risk behaviors may play a role in the clustering of cholera cases at very small (e.g., <100m) spatial scales. This had implications for spatially matched study designs, which may be overmatching on some exposures. It also may lead us to rethink targeted interventions, suggesting a role for more intensive highly targeted interventions as a supplement to more generalized campaigns.

## Introduction

Cholera is responsible for over 100,000 deaths each year and is endemic in many countries [1]. In Bangladesh, approximately 450,000 cases of cholera are estimated each year [2]. Cholera cases have been known to be tightly geographically clustered. For example, the scale of clustering of cholera cases in Matlab, Bangladesh was found to be around 250m in one study, and under 1km in another [3,4]. Close interpersonal contact likely drives clustering of secondary transmitted cases [5,6]. However, few studies have explored whether clustering in the risk factors themselves may explain clustering in disease.

Understanding if and how risk factors cluster may help us better understand how cholera and diarrheal diseases cluster. Furthermore, cholera prevention strategies often consist of behavioral interventions aimed at encouraging communities to adopt safe water, sanitation and hygiene practices [7]. The presence of micro-scale clustering would suggest that intensive, highly targeted, hygiene interventions might be effective complements to more general campaigns. Taking into account clustering of risk factors in modeling the effect of vaccine on cholera transmission can help identify optimal populations for vaccine deployment and thus increase effectiveness of immunization campaigns. More importantly, clustering of risk factors has important implications for the design of both spatially matched case-control studies and clustered survey design. Clustering of risk factors at a fine resolution may lead us to match on factors other than those we intended to match on when selecting spatially matched controls, biasing risk estimates towards the null and reducing the power of the study to detect associations [8]. It would also require cluster survey designs to have an increased sample size in order to account for correlation in risk, thus having profound implications for cholera vaccine studies adopting this design.

Here we use data from spatially matched households enrolled as controls during a case-control study to explore whether potential risk factors for cholera and other diarrheal diseases cluster at small spatial scales. The risk factors considered include lack of access to water, poor sanitation, overcrowding, source of drinking water, and poor hygiene and food handling practices [9–17]. These specific risk factors may or may not be responsible for cholera infection in the Arichpur neighborhood, but have been previously identified as important risk factors in Bangladesh and other countries [9–17]. We examine both the geographic clustering of individual risk factors, and whether groups of risk factors tend to appear together in individuals, households and nearby neighbors.

## Methods

### Study setting

The present study used data from households selected as spatially matched controls in a case-control study conducted between April and October 2013 in Arichpur in Tongi Township in Dhaka, Bangladesh. Arichpur is a cholera endemic working class neighborhood, and is approximately 1.2 km^2^ with a population density over 100,000 per km^2^ [18]. Cholera cases were consenting Arichpur residents aged 2 years or older who presented to local pharmacies or hospitals with acute diarrhea and had *V*. *Cholerae* O1 or O139 cultured from their stool sample. Hospital surveillance was conducted at the Tongi Hospital and the icddr,b Dhaka Hospital, both frequented by Arichpur residents.

### Selection of control households

Study staff attempted to enroll four spatially-matched control households (the matched-set) for each primary case household. To select a control household, staff would first select a number (one for each control household) from a random number table, then attempting to enroll the household that number of doors to the right of the entrance of the primary case household. Special instructions were applied for households in multi-story dwellings and when a natural boundary (e.g. railroad or river) was encountered. Household GPS locations were collected at the front door of each household (resolution ~3-5m).

### Data collection from control households

During one of the two household visits to each control household, we collected data on exposure to suspected cholera risk factors at both the household and individual level. Demographic variables including household size and number of rooms were collected. We also collected data on hygienic behavior including the availability of hand soap as observed by the team, and whether the household members boiled all of their (self-reported) sources of drinking water. Exposures related to sanitation and latrine included type of latrine and number of households sharing a latrine. For access to drinking water, we measured: 1) whether the household consumed drinking water from municipal supplied water (supplied water) and/or tubewell water in the past month by self-report; 2) distance to water source from front door of the house as measured by the team; 3) whether households had intermittent access to drinking water; and 4) whether and how households stored drinking water (See S1 Text for related questions from study questionnaire).

Individual-level exposures were collected at each visit from each consenting household member age 2 years of older. The exposures included: feeding a child by hand, eating meals prepared over 2 hours before consumption, drinking water outside the home, eating fresh cut fruit or vegetables outside the home, and drinking tea outside the home. The original questionnaire asked for frequency of each exposure in the past week (no exposure, 1–2 days, 3 or more days but not every day, or every day) (See S1 Text for related questions from study questionnaire).

### Modification of exposures for analysis

We dichotomized all exposures into high risk and low risk categories based on published literature on risk factors for cholera. Household density was defined as the ratio of number of household members to number of rooms, and was dichotomized at the mean. For other household level exposures, we determined that high risk categories were: using pit latrine (vs. modern/septic tank/sanitary), sharing a latrine with other households, storing drinking water, a distance of > 10 meters to nearest drinking water source from front door, intermittent drinking water supply, not always boiling drinking water from all reported source, no hand soap available, drinking municipal supplied water, and drinking tubewell water [9–17]. Although drinking municipal supplied water is a marker of improved water source under the Millennium Development Goals, we characterized it as a risk factor because the “improved sources” would still carry risks if treated inadequately [19–21]. Each individual level exposure was dichotomized into the exposed vs. not exposed categories. Exposures that showed little variability between households and individuals (or appeared in over 98% of households or individuals) were excluded from the main analyses (See S1 Text for details).

### Clustering of individual exposures

We explored the clustering of each household level exposure within matched-sets by calculating the intraclass correlation coefficient (ICC). For individual level exposures, we explored clustering within matched-sets as well as within individual households. We estimated the ICC using a two-level random effects logistic regression model with no additional covariates specified using the GLLAMM program in Stata 2012 [22,23]. We assumed a binomial distribution and specified logit link function for the binary exposures, representing the proportion of total variance in the underlying continuous latent response to each binary exposure that was due to the differences between matched-sets or households. We considered that the exposures with ICC estimates over 0.7 were strongly correlated, and the exposures with ICC estimates between 0.5 and 0.7 were moderately correlated.

### Microscale spatial dependence

To understand whether clustering of each household and individual level exposure exhibited fine scale spatial dependence, we explored the association between the level of clustering in matched-sets and their geographic extent. We quantified the clustering within each matched-set (within matched-set concordance) as the proportion of pairs of households (or individuals) within the matched-set that had the same exposure. We defined the spatial extent of matched-sets as the median distance between any two households within the matched-set. The confidence interval of the association was assessed using bootstrapping (1000 iterations) by resampling all matched-sets, households within the matched-sets, and then individuals within the households.

### Large-scale spatial dependence

We also explored whether clustering existed at larger spatial scales (i.e., distances greater than the spatial extent of matched-sets). We quantified concordance of exposures between two matched-sets (between matched-sets concordance) as the proportion of all possible pairs of households, one from each matched-set, that had the same exposure. We used non-parametric locally weighted polynomial regression models (LOESS) to visually assess how between matched-sets concordance for each exposure varied with the distance between centroids of matched-sets. We considered distances up to 780 meters (approximately ¾ of the diameter of Arichpur) as households and individuals that were separated by over 780 meters were limited. We hypothesized decreasing concordance between matched-sets for exposures that showed clustering beyond the spatial extent of the matched-sets. If concordance remained static beyond a certain distance threshold, the threshold likely corresponded to the spatial extent of the clustering. We used linear regressions to quantify the trend of decreasing concordance up to the threshold and calculated the confidence intervals using bootstrapping (1000 iterations) by resampling all matched-sets, households within the matched-sets, and then individuals within the households.

### Co-occurrence of different exposures

To understand which household level exposures clustered with one another, we calculated the probability of co-occurrence of different household level exposures within the same household, or matched-set. We then calculated the ratio of this probability to the probability of exposures appearing together by chance if they were uncorrelated. Likewise, we calculated the co-occurrence of each pair of individual level exposures within individuals, households and matched-sets (See S2 Text for detailed description of methods).

Values above one suggested two exposures tend to appear in the same individuals, households, or matched-sets closer to each other (co-occurrence of two exposures), while values less than one suggested two exposures tend to not appear together. Confidence intervals were calculated by resampling all matched-sets, households within the matched-sets, and then individuals within the households through 1000 bootstrap iterations.

### Ethics statement

We obtained written informed consent from adult study participants or parents/guardian for minors. The study protocol was reviewed and approved by the ethical review committee of Johns Hopkins Bloomberg School of Public Health and icddr,b.

## Results

**Fig 1:**
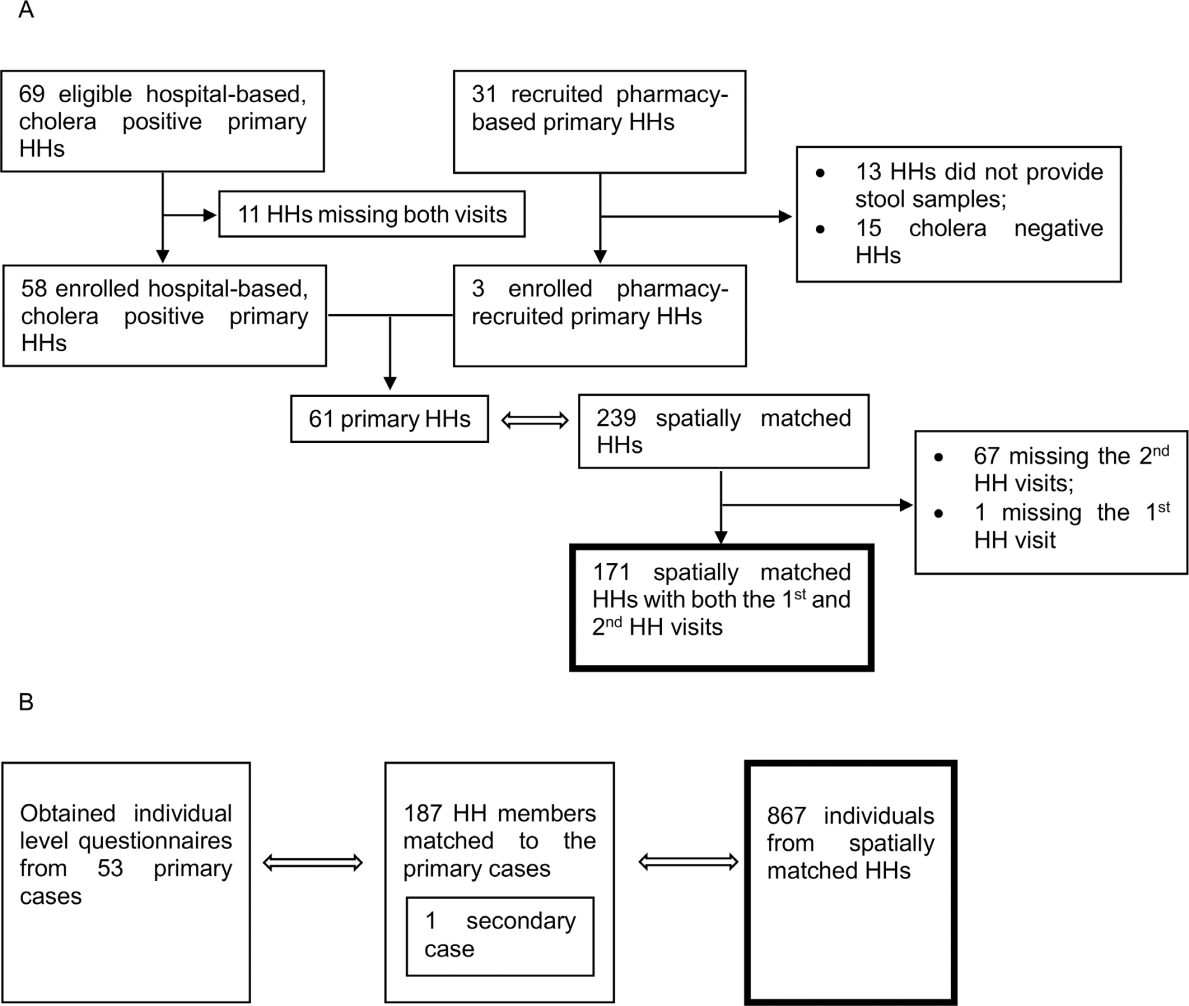
A flow chart outlining the enrolled households and individuals and those eligible for the study. A) For all household level exposures, only the data from the 171 spatially matched households participated in both household visits were considered. B) For analyses of individual level exposures, only the data from the 867 individuals in spatially matched households were considered.

Two hundred and thirty-nine households were recruited in 61 spatially matched-sets. Of those, 171 households (72%) in 48 matched-sets had complete household level exposure data, and were included in the present study (Fig 1). Eight hundred and sixty seven individuals from 239 households and 60 matched-sets were recruited, and were included for the individual level exposure analyses. The average geographic extent of the matched-sets was 42 meters (IQR = 22–55, range = 2–108), with an average of 3.6 households per matched-set (IQR = 3–4). We analyzed 10 household level and 5 individual level exposures from the spatially matched households (Table 1 and Table 2; See S1 Table for summary of exposures in the primary case households). We excluded one exposure that had almost no variability; 98.8% of households stored drinking water.

**Table 1:** The high risk categories of 10 exposures and the summary statistics. All household level exposures were dichotomized into high risk and low risk categories based on previous literature.

	Spatially Matched HHs with complete exposures (n = 171)	
Household Level Exposures	n	%
Household density above neighborhood average 3.2 ppl/room	65	38
Using pit latrine (vs. modern/septic tank/sanitary)	39	23
Sharing a latrine	151	88
Storing drinking water	169	99
Over 10 meters to the nearest drinking water source from front door	36	21
Intermittent drinking water supply	18	11
Consuming municipal supplied water in the past month	111	65
Consuming tubewell water in the past month	61	36
Not always boiling drinking water from all reported sources	114	68
Soap not available for handwashing	65	38

**Table 2:** Summary statistics of 5 individual level exposures. The individual level exposures were dichotomized to not exposed vs. exposed.

	Individuals in Matched Households (n = 867)	
Individual Level Exposures	n	%
Feeding a child with hand in the past week	203	28
Eating meals prepared over 2 hours before consumption in the past week	815	94
Drinking water outside the home in the past week	533	80
Eating fresh cut fruit or vegetables outside the home in the past week	103	17
Drinking tea outside the home in the past week	212	34

### Clustering of individual exposures

**Fig 2:**
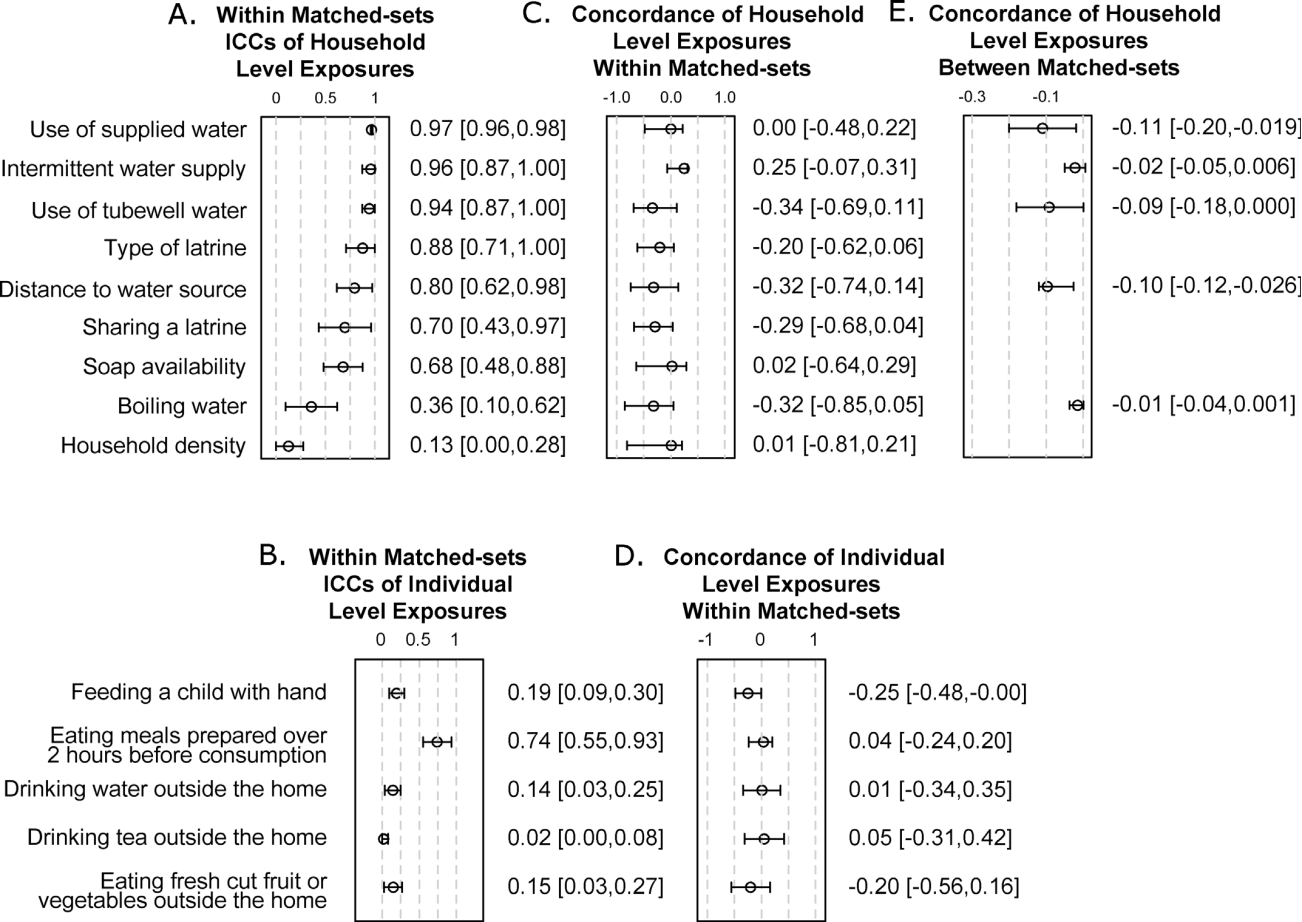
Clustering of individual exposures. A,B) Intraclass correlation coefficients (ICCs) and 95% confidence intervals of A) household level exposures and B) individual level exposures. All household level exposures except household density and boiling water showed clustering within matched-sets. For individual level exposures, only eating a meal prepared over 2 hours before consumption showed strong clustering. C,D) Linear association between the concordance of C) household level and D) individual level exposures within matched-sets and the spatial extent of matched-sets. Clustering within each matched-set (within matched-set concordance) was the proportion of pairs of households (or individuals) within the matched-set that had the same exposure. The spatial extent of matched-sets was the median distance between any two households within the matched-set. The coefficients and 95% confidence intervals specified the change in concordance per 100 meters increase in the spatial extent of matched-sets. More spatially compact matched-sets tended to have higher concordance in exposures than the larger ones, though the trend was not statistically significant for any exposure. E) Five exposures appeared to show clustering beyond the extent of matched-sets based on visual inspection of LOESS plots in Fig 3. The coefficients and 95% confidence intervals specified the change in concordance per 100 meters increase in the distance between matched-sets up to the spatial extent of clustering.

We observed pronounced clustering of household level exposures within matched-sets (Fig 2A). Neighboring households nearly always used the same type of drinking water (ICC for use of supplied water = 0.97, 95%CI = 0.96, 0.98; ICC for use of tubewell water = 0.94, 95%CI = 0.87, 1.00). Neighboring households frequently used the same type of latrine (ICC for modern vs. pit latrine = 0.88, 95%CI = 0.71, 1.00) and had similar access to drinking water (ICC intermittent water supply = 0.96, 95%CI = 0.87, 1.00, and distance to water source 0.80, 95%CI = 0.62, 0.98). Sharing a latrine (ICC = 0.70, 95%CI = 0.43, 0.97) and hygienic practices (ICC for soap availability = 0.68, 95%CI = 0.48, 0.88) showed moderate clustering. For individual behaviors, we only observed strong clustering of eating meals prepared over 2 hours before consumption (ICC = 0.90, 95% CI = 0.79, 1.00 within households; ICC = 0.74, 95% CI = 0.55, 0.93 for within matched-sets).

More spatially compact matched-sets tended to have higher concordance in exposures than the larger ones, though the trend was not statistically significant for any exposure (Fig 2C and 2D). Averaging the concordance of all exposures in each matched-set, we observed that, for each 100 meter increase in the geographic extent of a matched-set, the within matched-sets concordance decreased by 0.13 (95%CI = 0.064, 0.41) for household level exposures and by 0.069 (95%CI = -0.022, 0.19) for individual level exposures.

**Fig 3:**
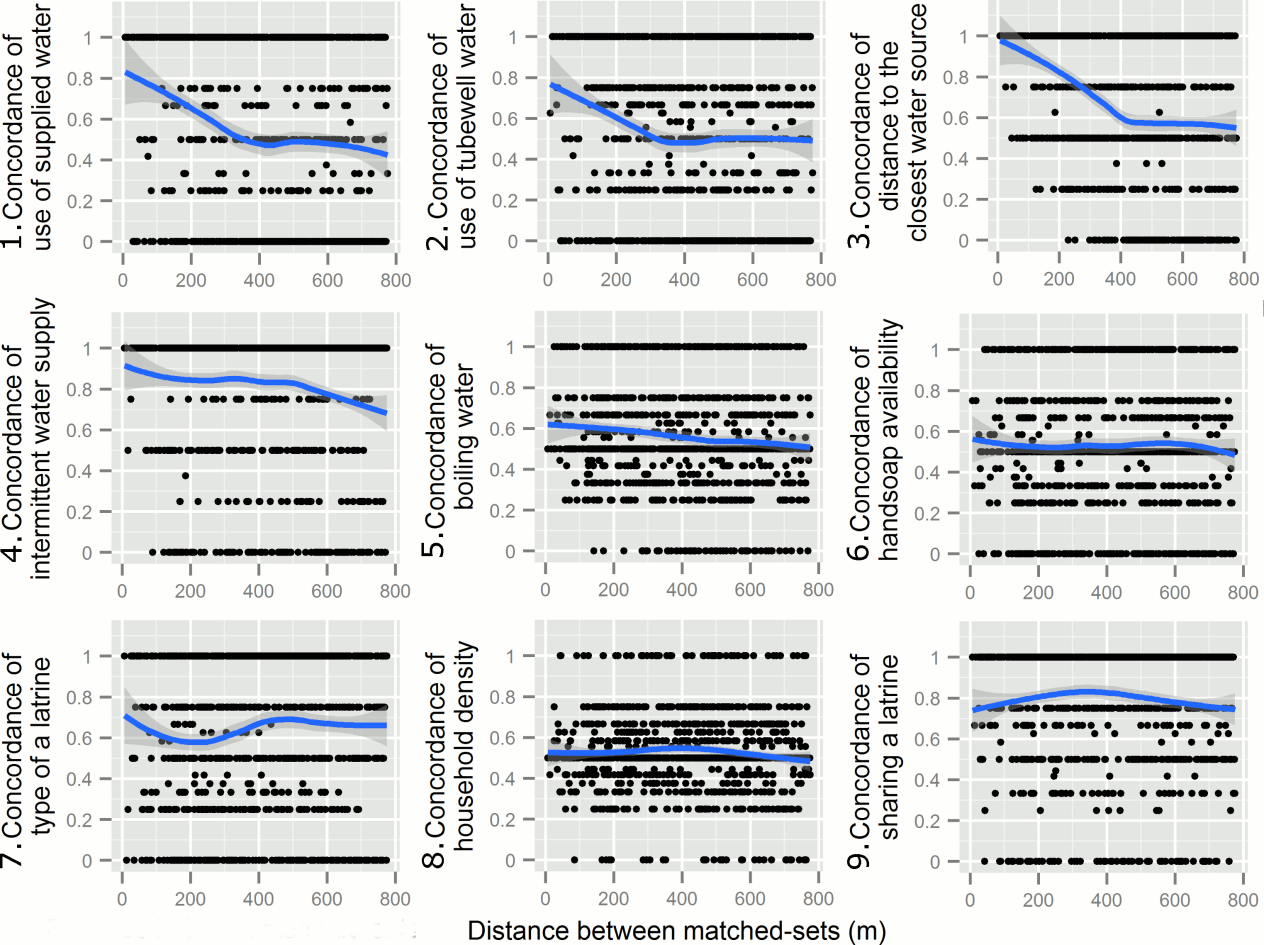
Clustering of exposures beyond the spatial extent of matched-sets. We visually assessed how between matched-sets concordance for each exposure varied with the distance between centroids of matched-sets using non-parametric locally weighted polynomial regression models (LOESS). The shaded area represented 95% confidence intervals of bootstrapped routine from 1,000 simulations. The first 5 exposures related to water source (Fig 3.1–5) showed decreasing concordance between matched-set over space, suggesting clustering of exposures beyond spatial extent of matched-set.

Visually assessing the LOESS plots of the exposures, we found clustering beyond the spatial extent of the matched-sets in household level exposures related to drinking water sources, as manifested by the decreasing concordance between matched-sets over space. The spatial extent of the clustering was approximately 350 meters for the use of supplied water and tubewell water, and 460 meters for distance over 10 meters to the closest water source (Fig 3), since concordance remained static beyond these thresholds. We also found an overall decreasing trend in concordance for intermittent water supply and boiling water. Considering distance up to the corresponding spatial extent of decreasing concordance, we observed that for each 100 meters increase in distances between two matched-sets, the concordance between matched-sets decreased by 0.11 (95%CI = 0.019, 0.20) for the use of supplied water, and 0.097 (95%CI = 0.026, 0.12) for the distance to the closest drinking water source. We did not observe strong decreasing concordance with distance in individual level exposures (S1 Fig).

### Co-occurrence of different exposures

**Fig 4:**
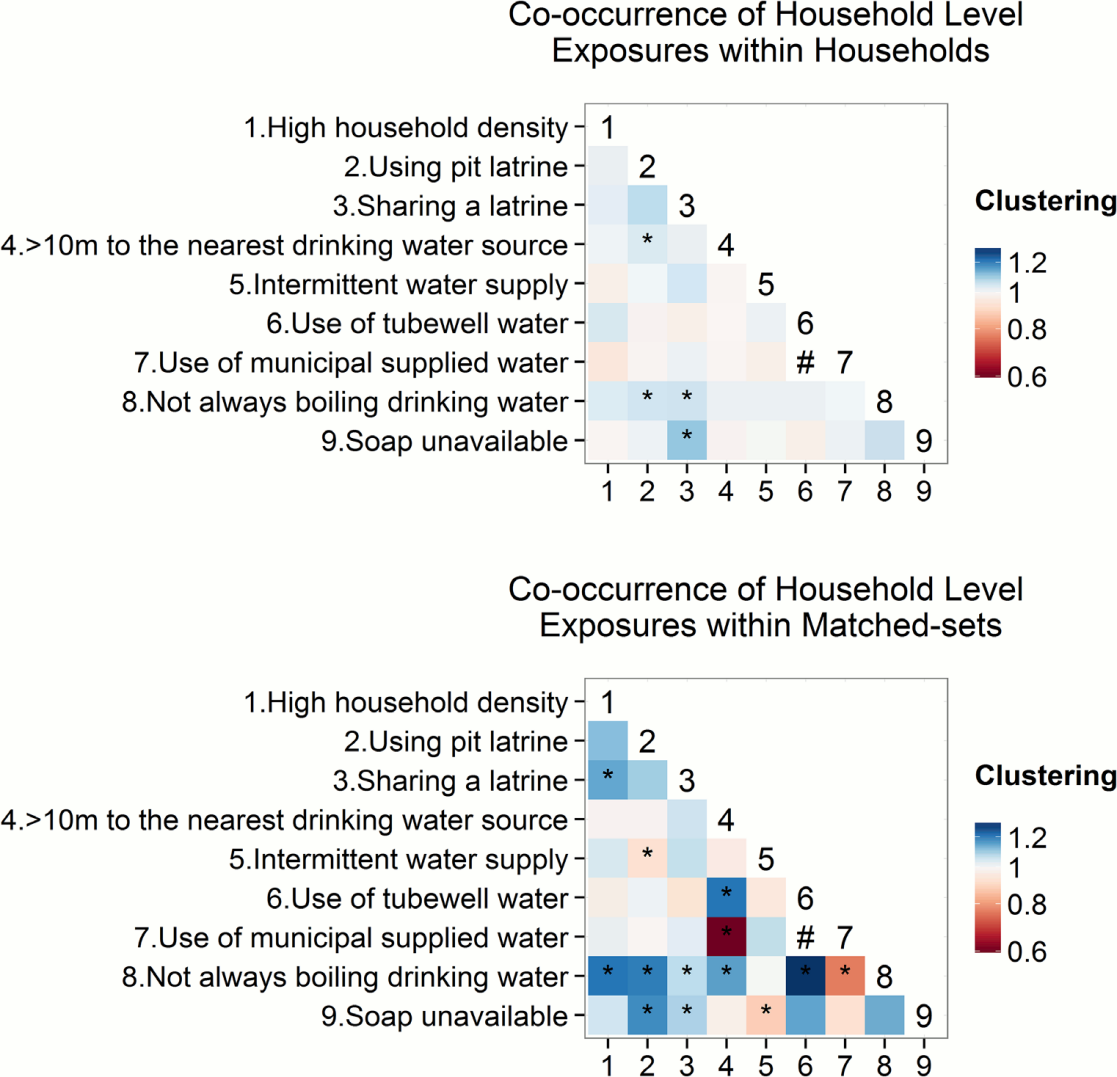
Co-occurrence of different household level exposures within households and within matched-sets. Values above one suggested two exposures tend to appear in the same households or matched-set closer to each other (co-occurrence of two exposures), while values less than one suggested two exposures tend to not appear together. Confidence interval was calculated by 1000 bootstrap iterations. Shading of each grid cell indicated the estimates of co-occurrence of different exposures, and the estimates that were statistically significant based on 1000 bootstrap iterations were marked by asteroids. Use of municipal supplied water and use of tubewell water was structurally correlated, thus the result was not shown in the graph and was marked by a #. As a result, when co-occurrence with the use of supplied water was over 1, co-occurrence with the use of tubewell water tended to be under 1. Household level unhygienic practices were correlated with infrastructure that is suspected to increase cholera risk. No pairs of exposures exhibited significant clustering in the same households.

Household level unhygienic practices were more common among households with infrastructure that was suspected to increase cholera risk. For example, households having no hand soap at home tended to tended to share a latrine with neighbors (1.11, 95%CI = 1.02, 1.19) (Fig 4A). Moreover, multiple risk factors related to water supply and sanitation infrastructure tended to exist in the same households; sharing a latrine was more common among households that were over 10m to the nearest drinking water source (1.04, 95%CI = 1.00, 1.08) (Fig 4A).

In addition, among the same matched-sets, unhygienic practices were correlated with infrastructure that was suspected to increase cholera risk. For example, households not always boiling drinking water tended to be in the same matched-set as those using a pit latrine (1.21, 95%CI = 1.07, 1.36) and those over 10m to the nearest water source (1.16, 95%CI = 1.07, 1.35) (Fig 4B; See S2 Table for co-occurrence of exposures with not boiling tubewell water and not boiling supplied water). Furthermore, the use of tubewell water and distance to water source were correlated in the same matched-sets (1.22, 95%CI = 1.06, 1.48) (Fig 4B). Clustering between individual level exposures appeared to mostly reflect the differing domestic roles of study participants, although the results were mostly statistically insignificant (S2 Fig).

## Discussion

We found substantial clustering of single exposures and co-occurrence of different exposures suspected to modify cholera and diarrhea risk within groups of nearby households, and an overall trend of households nearer to one another being more likely to share risk factors. This suggests that even within a relatively compact neighborhood there can be substantial spatial dependence of risk for cholera and diarrheal diseases driven by spatial homogeneity in behavior or access to improved water and sanitation.

Aggregating risk profiles at the neighborhood-level may be misleading and obfuscate hidden spatial heterogeneity, hence we may miss pockets of high risk. Thus, interventions targeted at micro-scale clusters of high cholera risk may be an important complement to general disease prevention or control campaigns. In cholera endemic neighborhoods, clusters of high-risk behavior could be identified prospectively through community surveys, or by canvasing the area around incident cholera cases. These groups could then be slated for more intensive water, sanitation, and hygienic interventions including education and provision of supplies. This latter course has proven effective in preventing onward spread of cholera in households, and may work equally well within clusters with high cholera risk [24].

Some study designs must account for spatial clustering of exposures at a fine scale. In a case control study, matching controls by proximity aims to control for environmental confounders that are unknown or difficult to measure, and is an efficient and practical method for risk factor studies or vaccine effectiveness studies in low-resource settings [25, 26]. However, if we do not increase sample size to account for the correlation in risk, spatially clustered designs will be underpowered to detect the effect of even non-clustered exposures, and this form of overmatching cannot be corrected in the analysis phase [8]. In our study, use of supplied water showed significant clustering within matched-sets, and hence was effectively matched on. If contaminated supplied water was associated with risk of cholera in Arichpur, we would be underpowered to detect a significant association unless we increased the sample size. Another study design, the cluster sample survey, has been widely used for estimating vaccination coverage and disease burden. In each cluster, households are usually selected based on their proximity to index households to reach the designated sampled size [27]. Clustering of risk factors increases design effect and our study has shown that it could be profound within a small community.

The study has several limitations. Our analysis focused only on one neighborhood in urban Bangladesh. Factors that are specific to the Arichpur neighborhood such as the network of water infrastructure affect the spatial scale of clustering. However, the result may likely be observed in other densely populated urban areas where cholera is most prevalent and similar risk profile is present. Thus, a clear understanding of risk factors present in the community is important for cholera interventions and studies. In addition, some exposures in our study are self-reported. Participants may be more likely to report low risk behavior, and may have undermined the level of clustering. However, the conclusion of microscale clustering would hold especially for the household level exposures, because they were less likely to be biased as infrastructure related to water supply and sanitation are very unlikely to change. Furthermore, 11 out of the 69 hospital-based cholera positive households did not participate in our study, and 13 out of the 31 households recruited from the pharmacy did not provide stool samples (Fig 1). If these missing households systematically differ from those in the study, our results may be biased.

In conclusion, our study demonstrates clustering of individual and groups of exposures modifying risk of cholera and diarrheal diseases within immediate neighbors, and an overall trend of clustering over the entire Arichpur neighborhood. Based on our findings on clustering of high-risk behaviors, we recommend incorporating community risk assessments to supplement general water, sanitation, and hygienic intervention campaigns. For clustered studies such as those designed to assess vaccine effectiveness and identify risk factors, we call for an initial risk assessment for level of risk factor clustering, and determine the required sample size accordingly.

## Supporting Information

S1 Fig Clustering of each individual level exposures beyond the spatial extent of matched-sets. (TIFF)

S2 Fig Co-occurrence of individual level exposures. (TIFF)

S1 Text Original survey questions related to risk factors included in this study. (DOCX)

S2 Text Methods for calculating co-occurrence of exposures. (DOCX)

S1 Table Summary statistics of 10 household level exposures among primary households, and 5 individual level exposures among negative individuals in the primary households. (DOCX)

S2 Table Co-occurrence of household level risk factors with two additional risk factors, not boiling tubewell water and not boiling municipal supplied water. (DOCX)

S1 Checklist STROBE checklist. (DOC)
