# Supplementary material for: Micro-scale Spatial Clustering of Cholera Risk Factors in Urban Bangladesh
Source: PLoS Negl Trop Dis. 2016 Feb 11;10(2):e0004400. doi: 10.1371/journal.pntd.0004400 (PMC4750854; doi:10.1371/journal.pntd.0004400)
Supplement: S1 Text — (DOCX) [file pntd.0004400.s003.docx]

**Investigating the transmission of cholera in Arichpur, Tongi:**

**A pilot study**

**Household Questionnaire**

1. **How many people currently live in this household (or dormitory room)?**

*A household is defined as a person or group of related and/or unrelated persons who usually live in the same dwelling unit(s), who have common cooking and eating arrangements, and who acknowledge one adult member as head of household. A member of the household is any person who usually lives in the household.*

*In the case of a dormitory, household refers to those living in the same room as the individual and the household head is defined as the first eligible consenting adult in this study.*

☐ *unsure* ☐ *decline*

1. **How many rooms in your current house?**

*(A room is defined as an area with one or more doors or entrance ways and otherwise surrounded by walls. In multi-family households, only count rooms used or shared by members of the household. If dormitory, the number of rooms where people sleep in the entire structure.)*

*decline* ☐

1. **Do you have any soap or detergent available for hand washing?** *(Observe)*

*If yes, ask to see the soap or detergent.*

*If the soap or detergent is not available within 45 seconds, mark “no”*

☐ yes ☐ *decline*

☐ no

1. **Now I am going to ask you a series of questions about the sources of drinking water in your household and things that people in your household do to water before they drink.**

*For each source, ask each question across the columns. If the answer to 5 for any source is “no”, do not proceed to additional questions about that source. These questions are about use of water within the household.*

|  | 1. **Used in the past month?** | 1. **Boil before drinking water from…?**   *(only answer for those sources with a yes in 2.1)* |
| --- | --- | --- |
| **Supply** | ☐ **yes**  ☐ **no**  *unknown* ☐  *decline* ☐ | ☐ **always**  ☐ **sometimes**  ☐ **never**  *unknown* ☐  *decline* ☐ |
| **Tube well/**  **Borehole** | ☐ **yes**  ☐ **no**  *unknown* ☐  *decline* ☐ | ☐ **always**  ☐ **sometimes**  ☐ **never**  *unknown* ☐  *decline* ☐ |

**Water and Sanitation Assesment**

1. **Can you show me where people in your household usually defecate?** *(Observe)*

☐ open place ☐ *decline*

☐ hanging/open latrine

☐ pit latrine without/broken slab

☐ pit latrine with slab but no water seal

☐ pit latrine with slab & water seal

☐ modern/septic tank/sanitary

☐ other (specify) _________________`

1. **How many households, including yours, share this defecation area?**

☐ only this household ☐ *decline*

☐ 2

☐ 3-5

☐ >5

1. **Do you store drinking water in your household before drinking?**

☐ yes, store in vessel with tap ☐ *decline*

☐ yes, store in vessel with no tap

☐ no, does not store water in household

1. **How often is water available from this water source?**

☐ always available ☐ *decline*

☐ intermittent

1. **Note approximately how far is this water source is from the front door of the house.**

☐ inside household ☐ *decline*

☐ less than 10 meters

☐ more than 10 meters

**Individual Questionnaire**

1. **In the past week how often did you feed a child with your hand?**

☐ did not do this

☐ one to two days

☐ three or more days but not everyday

☐ everyday

*decline* ☐

1. **In the past week did how often did you eat a meal that was prepared more than 2 hours before you ate it?** *(🡪 skip to 40)*

☐ did not do this

☐ one to two days

☐ three or more days but not everyday

☐ everyday

*unknown* ☐ *decline* ☐

1. **In the past week on how many days did you drink water at the following locations?**

| **Home** | **Work/School** | **Elsewhere** |
| --- | --- | --- |
| ☐ **did not do this**  ☐ **one to two days**  ☐ **three or more days but not everyday**  ☐ **everyday**  *decline* ☐ | ☐ **did not do this**  ☐ **one to two days**  ☐ **three or more days but not everyday**  ☐ **everyday**  *decline* ☐ | ☐ **did not do this**  ☐ **one to two days**  ☐ **three or more days but not everyday**  ☐ **everyday**  *decline* ☐ |

1. **In the past week on how many days did you drink/eat the following items?**

*For each row (type of food/drink) ask how often they consumed it in each location (column). For example you would first ask, “In the past week how many days did you drink fresh fruit juice at home?”*

|  | **Home** | **Work or School** | **Elsewhere** |
| --- | --- | --- | --- |
| 1. **Fresh cut fruit or vegetables** | ☐ **did not do this**  ☐ **one to two days**  ☐ **three or more days but not everyday**  ☐ **everyday**  *decline* ☐ | ☐ **did not do this**  ☐ **one to two days**  ☐ **three or more days but not everyday**  ☐ **everyday**  *decline* ☐ | ☐ **did not do this**  ☐ **one to two days**  ☐ **three or more days but not everyday**  ☐ **everyday**  *decline* ☐ |
| 1. **Tea** | ☐ **did not do this**  ☐ **one to two days**  ☐ **three or more days but not everyday**  ☐ **everyday**  *decline* ☐ | ☐ **did not do this**  ☐ **one to two days**  ☐ **three or more days but not everyday**  ☐ **everyday**  *decline* ☐ | ☐ **did not do this**  ☐ **one to two days**  ☐ **three or more days but not everyday**  ☐ **everyday**  *decline* ☐ |
