# Supplementary material for: Micro-scale Spatial Clustering of Cholera Risk Factors in Urban Bangladesh
Source: PLoS Negl Trop Dis. 2016 Feb 11;10(2):e0004400. doi: 10.1371/journal.pntd.0004400 (PMC4750854; doi:10.1371/journal.pntd.0004400)
Supplement: S2 Text — (DOCX) [file pntd.0004400.s004.docx]

**S2 Text. Methods for calculating co-occurrence of household level and individual level exposures.**

**Household level exposures**

We calculated the probability of co-occurrence of different household level exposures within the same household, the same matched-set, and the Arichpur neighborhood as follows:

$\boldsymbol{Pr}\boldsymbol{(}\boldsymbol{z}_{\boldsymbol{i}\boldsymbol{,}\boldsymbol{A}}\boldsymbol{=}\boldsymbol{z}_{\boldsymbol{i,}\boldsymbol{B}}\boldsymbol{)}$**,** within the same households

$\boldsymbol{Pr}\left( \boldsymbol{z}_{\boldsymbol{i,A}}\boldsymbol{=}\boldsymbol{z}_{\boldsymbol{j\neq i, B}} \right|\boldsymbol{j}\boldsymbol{\in\Omega}_{\boldsymbol{i}}\boldsymbol{)}$**,** within the same matched-sets

$\boldsymbol{Pr}\boldsymbol{(}\boldsymbol{z}_{\boldsymbol{i}\boldsymbol{,}\boldsymbol{A}}\boldsymbol{=}\boldsymbol{z}_{\boldsymbol{j\neq i}\boldsymbol{,}\boldsymbol{B}}\boldsymbol{)}$**,** within the Arichpur neighborhood

Where $\boldsymbol{z}_{\boldsymbol{i}\boldsymbol{,}\boldsymbol{A}}\boldsymbol{=}\boldsymbol{z}_{\boldsymbol{i,}\boldsymbol{B}}$ when a household *i* had exposure to both risk factors *A* and *B*, $\boldsymbol{z}_{\boldsymbol{i,A}}\boldsymbol{=}\boldsymbol{z}_{\boldsymbol{j\neq i, B}}$ when risk factor *A* from household *i* was concordant with risk factor *B* from another household *j*, and $\boldsymbol{\Omega}_{\boldsymbol{i}}$ represented the set of households in the same matched-set as household *i*.

To account for the probability of exposures appearing together by chance, we calculated the relative probability of co-occurrence of difference risk factor appearing in the same household compared with the probability of co-occurrence of risk factor appearing between households in the same matched-set as follows:

Co-occurrence of different exposures within the same households accounting for chance

$$\boldsymbol{=}\frac{\boldsymbol{Pr}\boldsymbol{(}\boldsymbol{z}_{\boldsymbol{i}\boldsymbol{,}\boldsymbol{A}}\boldsymbol{=}\boldsymbol{z}_{\boldsymbol{i,}\boldsymbol{B}}\boldsymbol{)}}{\boldsymbol{Pr}\left( \boldsymbol{z}_{\boldsymbol{i,A}}\boldsymbol{=}\boldsymbol{z}_{\boldsymbol{j\neq i, B}} \right|\boldsymbol{j}\boldsymbol{\in\Omega}_{\boldsymbol{i}}\boldsymbol{)}}$$

Similarly, we calculated co-occurrence of different exposures within the same matched-sets accounting for chance as

$$\boldsymbol{=}\frac{\boldsymbol{Pr}\left( \boldsymbol{z}_{\boldsymbol{i,A}}\boldsymbol{=}\boldsymbol{z}_{\boldsymbol{j\neq i,B}} \right|\boldsymbol{j}\boldsymbol{\in\Omega}_{\boldsymbol{i}}\boldsymbol{)}}{\boldsymbol{Pr}\boldsymbol{(}\boldsymbol{z}_{\boldsymbol{i}\boldsymbol{,}\boldsymbol{A}}\boldsymbol{=}\boldsymbol{z}_{\boldsymbol{j\neq i}\boldsymbol{,}\boldsymbol{B}}\boldsymbol{)}}$$

The significance of clustering of risk factor pairs was assessed by 1000 bootstrap iterations, where both matched-sets and households were sampled with replacement.

**Individual level exposures**

Similarly, we calculated the probability of co-occurrence of different individual level exposures within the same individual, household, matched-set, and the Arichpur neighborhood as follows:

$\boldsymbol{Pr}\boldsymbol{(}\boldsymbol{z}_{\boldsymbol{i}\boldsymbol{,}\boldsymbol{A}}\boldsymbol{=}\boldsymbol{z}_{\boldsymbol{i,}\boldsymbol{B}}\boldsymbol{)}$**,** within the same individual

$\boldsymbol{Pr}\left( \boldsymbol{z}_{\boldsymbol{i,A}}\boldsymbol{=}\boldsymbol{z}_{\boldsymbol{j\neq i, B}} \right|\boldsymbol{j}\boldsymbol{\in\varphi}_{\boldsymbol{i}}\boldsymbol{)}$, within the same household

$\boldsymbol{Pr}\left( \boldsymbol{z}_{\boldsymbol{i,A}}\boldsymbol{=}\boldsymbol{z}_{\boldsymbol{j\neq i, B}} \right|\boldsymbol{j}\boldsymbol{\in\Omega}_{\boldsymbol{i}}\boldsymbol{)}$, within the same matched-set

$\boldsymbol{Pr}\boldsymbol{(}\boldsymbol{z}_{\boldsymbol{i}\boldsymbol{,}\boldsymbol{A}}\boldsymbol{=}\boldsymbol{z}_{\boldsymbol{j\neq i}\boldsymbol{,}\boldsymbol{B}}\boldsymbol{)}$**,** within the Arichpur neighborhood

where $\boldsymbol{z}_{\boldsymbol{i}\boldsymbol{,}\boldsymbol{A}}\boldsymbol{=}\boldsymbol{z}_{\boldsymbol{i,}\boldsymbol{B}}$ when the same individual had exposure to both risk factors *A* and *B*, $\boldsymbol{z}_{\boldsymbol{i,A}}\boldsymbol{=}\boldsymbol{z}_{\boldsymbol{j\neq i, B}}$ when risk factor *A* from individual *i* was concordant with risk factor *B* from another individual *j*, $\boldsymbol{\varphi}_{\boldsymbol{i}}$ represented the household members of *i*, and $\boldsymbol{\Omega}_{\boldsymbol{i}}$ represented the set of individuals in the same matched-set as individual *i*.

To account for the probability of exposures appearing together by chance, we calculated the relative probability of co-occurrence of difference risk factor appearing in the same individual compared with the probability of co-occurrence of risk factor appearing between individuals in the same household as follows:

Co-occurrence of different exposures within the same individuals accounting for chance as

$$\boldsymbol{=}\frac{\boldsymbol{Pr}\boldsymbol{(}\boldsymbol{z}_{\boldsymbol{i}\boldsymbol{,}\boldsymbol{A}}\boldsymbol{=}\boldsymbol{z}_{\boldsymbol{i,}\boldsymbol{B}}\boldsymbol{)}}{\boldsymbol{Pr}\left( \boldsymbol{z}_{\boldsymbol{i,A}}\boldsymbol{=}\boldsymbol{z}_{\boldsymbol{j\neq i, B}} \right|\boldsymbol{j}\boldsymbol{\in\varphi}_{\boldsymbol{i}}\boldsymbol{)}}$$

Similarly, we calculated co-occurrence of different exposures within the same households accounting for chance as

$$\boldsymbol{=}\frac{\boldsymbol{Pr}\left( \boldsymbol{z}_{\boldsymbol{i,A}}\boldsymbol{=}\boldsymbol{z}_{\boldsymbol{j\neq i, B}} \right|\boldsymbol{j}\boldsymbol{\in\varphi}_{\boldsymbol{i}}\boldsymbol{)}}{\boldsymbol{Pr}\left( \boldsymbol{z}_{\boldsymbol{i,A}}\boldsymbol{=}\boldsymbol{z}_{\boldsymbol{j\neq i, B}} \right|\boldsymbol{j}\boldsymbol{\in\varphi}_{\boldsymbol{i}}\boldsymbol{)}}$$

and the co-occurrence of different exposures within the same matched-sets accounting for chance as

$$\boldsymbol{=}\frac{\boldsymbol{Pr}\left( \boldsymbol{z}_{\boldsymbol{i,A}}\boldsymbol{=}\boldsymbol{z}_{\boldsymbol{j\neq i,B}} \right|\boldsymbol{j}\boldsymbol{\in\Omega}_{\boldsymbol{i}}\boldsymbol{)}}{\boldsymbol{Pr}\boldsymbol{(}\boldsymbol{z}_{\boldsymbol{i}\boldsymbol{,}\boldsymbol{A}}\boldsymbol{=}\boldsymbol{z}_{\boldsymbol{j\neq i}\boldsymbol{,}\boldsymbol{B}}\boldsymbol{)}}$$

The significance of clustering of risk factor pairs was assessed by 1000 bootstrap iterations, where matched-sets, households, and individuals were sampled with replacement.
