# Supplementary material for: Micro-scale Spatial Clustering of Cholera Risk Factors in Urban Bangladesh
Source: PLoS Negl Trop Dis. 2016 Feb 11;10(2):e0004400. doi: 10.1371/journal.pntd.0004400 (PMC4750854; doi:10.1371/journal.pntd.0004400)
Supplement: S1 Table — (DOCX) [file pntd.0004400.s005.docx]

S1 Table A. Summary statistics of 10 household level exposures among primary households. All household level exposures were dichotomized into high risk and low risk categories based on previous literature.

|  | **Primary households with complete exposures (n=41)** | |
| --- | --- | --- |
| **High Risk Categories of Household Level Exposures** | **n** | **%** |
| Household density above 3.2 ppl/room | 18 | 44 |
| Using pit latrine (vs. modern/septic tank/sanitary) | 12 | 29 |
| Sharing a latrine | 37 | 90 |
| Storing drinking water | 41 | 100 |
| Over 10 meters to the nearest drinking water source from front door | 8 | 20 |
| Intermittent drinking water supply | 3 | 7 |
| Consuming municipal supplied water in the past month | 33 | 80 |
| Consuming tube water in the past month | 12 | 29 |
| Not always boiling drinking water | 35 | 85 |
| Soap not available for handwashing | 17 | 41 |

S1 Table B. Summary statistics of 5 individual level exposures among negative individuals in the primary households. All individual level exposures were dichotomized to not exposed vs. exposed categories.

|  | **Cholera negative individuals in primary households (n=187)** | |
| --- | --- | --- |
| **High Risk Categories of Individual Level Exposures** | **n** | **%** |
| Feeding a child with hand in the past week | 33 | 20 |
| Eating meals prepared over 2 hours before consumption in the past week | 172 | 92 |
| Drinking water outside the home in the past week | 128 | 84 |
| Eating fresh cut fruit or vegetables outside the home in the past week | 24 | 17 |
| Drinking tea outside the home in the past week | 61 | 41 |
