# Supplementary material for: Micro-scale Spatial Clustering of Cholera Risk Factors in Urban Bangladesh
Source: PLoS Negl Trop Dis. 2016 Feb 11;10(2):e0004400. doi: 10.1371/journal.pntd.0004400 (PMC4750854; doi:10.1371/journal.pntd.0004400)
Supplement: S2 Table — (DOCX) [file pntd.0004400.s006.docx]

S2 Table. Co-occurrence of household level risk factors with two additional risk factors, not boiling tubewell water and not boiling municipal supplied water.

|  | Not boiling tubewell water (n=54) ᵃ | | Not boiling municipal supplied water (n=63) ᵇ | |
| --- | --- | --- | --- | --- |
|  | Within households | Within matched-sets | Within households | Within matched-sets |
| Household density | 1.06[0.98,1.13] | 1.14[0.98,1.27] | 1.00[0.89,1.07] | 1.23[1.01,1.34] |
| Using pit latrine | 0.92[0.84,1.35] | 1.17[0.86,1.35] | 1.06[0.98,1.13] | **1.27[1.10,1.51]** |
| Sharing a latrine | 1.07[0.96,1.21] | **1.13[1.02,1.28]** | **1.07[1.00,1.20]** | 1.04[0.96,1.15] |
| Storing drinking water | 0.97[0.88,1.06] | 1.10[0.97,1.34] | 0.99[0.92,1.06] | 1.05[0.91,1.17] |
| Over 10 meters to the nearest drinking water source from front door | 0.93[0.83,1.34] | 1.19[0.76,1.33] | 1.00[0.94,1.11] | 1.03[0.88,1.21] |
| Intermittent drinking water supply | 1.01[0.97,1.03] | 0.99[0.97,1.03] | 0.97[0.91,1.04] | 1.04[0.93,1.08] |

ᵃ 57% of households that used tubewell water always boiled the water

ᵇ 89% of households that used municipal supplied water always boiled the water
