# Supplementary material for: Micro-scale Spatial Clustering of Cholera Risk Factors in Urban Bangladesh
Source: PLoS Negl Trop Dis. 2016 Feb 11;10(2):e0004400. doi: 10.1371/journal.pntd.0004400 (PMC4750854; doi:10.1371/journal.pntd.0004400)
Supplement: S1 Checklist — (DOC) [file pntd.0004400.s007.doc]

STROBE Statement—Checklist of items that should be included in reports of ***case-control studies***

|  | Item No | Recommendation |
| --- | --- | --- |
| **Title and abstract** | 1 | (*a*) Indicate the study’s design with a commonly used term in the title or the abstract  **see lines 18-20** |
|  |  | (*b*) Provide in the abstract an informative and balanced summary of what was done and what was found  **see lines 18-21** |
| Introduction | | |
| Background/rationale | 2 | Explain the scientific background and rationale for the investigation being reported  **see lines 48-54** |
| Objectives | 3 | State specific objectives, including any prespecified hypotheses  **see lines 56-69** |
| Methods | | |
| Study design | 4 | Present key elements of study design early in the paper  **see lines 82-118** |
| Setting | 5 | Describe the setting, locations, and relevant dates, including periods of recruitment, exposure, follow-up, and data collection  **see lines 82-118** |
| Participants | 6 | (*a*) Give the eligibility criteria, and the sources and methods of case ascertainment and control selection. Give the rationale for the choice of cases and controls  **see lines 82-98** |
|  |  | (*b*) For matched studies, give matching criteria and the number of controls per case  **see lines 92-98** |
| Variables | 7 | Clearly define all outcomes, exposures, predictors, potential confounders, and effect modifiers. Give diagnostic criteria, if applicable  **see lines 85-113. We did not perform risk factor analysis.** |
| Data sources/ measurement | 8* | For each variable of interest, give sources of data and details of methods of assessment (measurement). Describe comparability of assessment methods if there is more than one group  **see lines 120-133** |
| Bias | 9 | Describe any efforts to address potential sources of bias  **see lines 135-184** |
| Study size | 10 | Explain how the study size was arrived at  **see lines 86-93** |
| Quantitative variables | 11 | Explain how quantitative variables were handled in the analyses. If applicable, describe which groupings were chosen and why  **see lines 101-133** |
| Statistical methods | 12 | (*a*) Describe all statistical methods, including those used to control for confounding  **This section is not relevant to our statistical methods, as this paper examines spatial clustering of risk factors. See lines 135-184 for our detailed statistical methods.** |
|  |  | (*b*) Describe any methods used to examine subgroups and interactions  **This section is not relevant to our statistical methods, as this paper examines spatial clustering of risk factors. See lines 135-184 for our detailed statistical methods.** |
|  |  | (*c*) Explain how missing data were addressed  **This section is not relevant to our statistical methods, as this paper examines spatial clustering of risk factors. See lines 135-184 for our detailed statistical methods.** |
|  |  | (*d*) If applicable, explain how matching of cases and controls was addressed  **This section is not relevant to our statistical methods, as this paper examines spatial clustering of risk factors. See lines 135-184 for our detailed statistical methods.** |
|  |  | (*e*) Describe any sensitivity analyses  **This section is not relevant to our statistical methods, as this paper examines spatial clustering of risk factors. See lines 135-184 for our detailed statistical methods.** |
| Results | | |
| Participants | 13* | (a) Report numbers of individuals at each stage of study—eg numbers potentially eligible, examined for eligibility, confirmed eligible, included in the study, completing follow-up, and analysed  **See lines 191-195, 200-203** |
|  |  | (b) Give reasons for non-participation at each stage  **See lines 191-195, 200-203** |
|  |  | (c) Consider use of a flow diagram  **See lines 200-203, Figure 1** |
| Descriptive data | 14* | (a) Give characteristics of study participants (eg demographic, clinical, social) and information on exposures and potential confounders  **see lines 205-210 for characteristics of study participants. Information on potential confounders is not relevant.** |
|  |  | (b) Indicate number of participants with missing data for each variable of interest  **see lines 200-210.** |
| Outcome data | 15* | Report numbers in each exposure category, or summary measures of exposure  **see lines 205-210** |
| Main results | 16 | (*a*) Give unadjusted estimates and, if applicable, confounder-adjusted estimates and their precision (eg, 95% confidence interval). Make clear which confounders were adjusted for and why they were included  **Unadjusted/adjusted estimates are not relevant.** |
|  |  | (*b*) Report category boundaries when continuous variables were categorized  **Category boundaries are not relevant.** |
|  |  | (*c*) If relevant, consider translating estimates of relative risk into absolute risk for a meaningful time period  **Estimates of relative risk are not relevant.** |

| Other analyses | 17 | Report other analyses done—eg analyses of subgroups and interactions, and sensitivity analyses  **Analysis of interactions is not relevant.** |
| --- | --- | --- |
| Discussion | | |
| Key results | 18 | Summarise key results with reference to study objectives  **See lines 299-301** |
| Limitations | 19 | Discuss limitations of the study, taking into account sources of potential bias or imprecision. Discuss both direction and magnitude of any potential bias  **See lines 331-343** |
| Interpretation | 20 | Give a cautious overall interpretation of results considering objectives, limitations, multiplicity of analyses, results from similar studies, and other relevant evidence  **See lines 301-304** |
| Generalisability | 21 | Discuss the generalisability (external validity) of the study results  **See lines 331-335** |
| Other information | | |
| Funding | 22 | Give the source of funding and the role of the funders for the present study and, if applicable, for the original study on which the present article is based  **Included relevant information under the Funding Information** |

*Give information separately for cases and controls.

**Note:** An Explanation and Elaboration article discusses each checklist item and gives methodological background and published examples of transparent reporting. The STROBE checklist is best used in conjunction with this article (freely available on the Web sites of PLoS Medicine at http://www.plosmedicine.org/, Annals of Internal Medicine at http://www.annals.org/, and Epidemiology at http://www.epidem.com/). Information on the STROBE Initiative is available at http://www.strobe-statement.org.
